# Disinfection of Dental Unit Water Line Using Aloe Vera: In Vitro Study

**DOI:** 10.1155/2013/618962

**Published:** 2013-09-08

**Authors:** Sonia Pareek, Anup Nagaraj, Prateek Sharma, Mansi Atri, Satinder Walia, Shravani Naidu, Asif Yousuf

**Affiliations:** ^1^Public Health Dentistry, Jaipur Dental College, Dhand-AmerJaipur Delhi, NH-8, Jaipur 303101, India; ^2^Comprehensive Healthcare, Jaipur 302001, India; ^3^Public Health Dentistry, I.T.S. Dental College, Noida 201308, India; ^4^Public Health Dentistry, Sri Guru Ram Das Institute of Dental Science, Amritsar 143006, India

## Abstract

*Context*. Dental unit waterlines may be heavily contaminated with microorganisms and are a potential source of infection for both practicing staff and immunocompromised patients particularly. Contamination of dental unit water lines could be inhibited with the use of disinfectants. The present study investigates the effect of aloe-vera-based disinfectant in reducing the microbial growth in dental unit water lines (DUWLs). *Aims*. To compare the efficacy of aloe vera, hydrogen peroxide (H_2_O_2_), and 5% sodium hypochlorite (NaOCl) in controlling microbial contamination of DUWLs. *Materials and Methods*. After obtaining baseline water samples, the dental unit waterlines were treated with aloe vera, 10% hydrogen peroxide, and 5% sodium hypochlorite. Each of the three disinfectants was used in increasing concentrations and their inhibiting effect was compared. Water samples were analyzed for microbiological quality by the total viable count (TVC) method. *Statistical Analysis Used*. SPSS 16. *Results*. There was significant reduction in mean CFU/ml when treated with disinfectants each for a period of one week. Aloe-vera solution was found to be the most effective in reducing the microbial colonies. *Conclusions*. Improving the water quality from dental unit water lines is of considerable importance; chemical-based disinfectants can be replaced with herbal disinfectants for treating microbial contamination in dental unit waterlines.

## 1. Introduction

The term “biofilm” refers to assemblage of microbial cells, that is, irreversibly associated with a surface and enclosed in a matrix of primarily polysaccharide material. The growth of biofilm is considered to be a result of complex processes involving transport of organic and inorganic molecules and microbial cells to the surface, adsorption of molecules to the surface, and initial attachment of microbial cells followed by their irreversible adhesion, facilitated by production of extracellular polymeric substances, often referred to as glycocalyx or slime [1].

Organisms in dental unit water line biofilm are predominantly derived from the incoming mains water. Once a new DUWL system is connected to the mains water supply, a biofilm will form within eight hours. Biofilm formation in dental unit water lines takes place even when it is not used for treatment of patients [2]. The nature of DUWLs is such that they will develop a biofilm, and water flowing down the biofilm coated waterlines will contribute to microbial load in the water as it exits the tubing. Frequent periods of water stagnation in DUWLs and the properties of the plastics used in DUWLs construction can promote the attachment and colonization of biofilm forming microorganisms [3].

To keep the biofilm and the contamination of the DUWLs under control, or rather to reduce the risk of infection both for the patients and for the health staff, use of water-line flushing, independent water reservoir systems, distilled or pasteurized water, ultrasonic, ultraviolet light, inline micropore filtration, and periodic or continuous chemical disinfection has been suggested [4]. 

The efficacy of disinfection is affected by a number of factors, including the prior cleaning of the object, the organic load present, the type and level of microbial contamination, the concentration of and exposure time to the germicide, the nature of the object, and the temperature and pH of the disinfection process. Disinfectants may be further subdivided by their efficacy. A few disinfectants will kill endospores after prolonged exposure times (i.e., 6 to 10 hours) and are called chemical sterilants [5].

Biofilms are emerging as an increasing problem as medical technology advances. Dental practice has no exception and interest in the role of biofilms within dental units as a possible source of cross infection is intensifying. It is difficult to quantitate the risks associated with aerosolised bacteria for the majority of patients seen in general practice. However, it seems prudent to eliminate this source of infection during treatment of compromised patients. In search of a practical solution to reduce microbial contamination of DUWLs, the present study is conducted to test the efficacy of aloe vera solution to decrease the number of bacteria in DUWLs.

## 2. Aim

To compare the efficacy of aloe vera, 5% sodium hypochlorite (NaOCl), and 10% hydrogen peroxide (H_2_O_2_), each at different concentrations in controlling microbial contamination of dental unit water systems.

## 3. Objectives

- To compare the efficacy of aloe vera, 5% NaOCl, and 10% hydrogen peroxide as disinfectants for dental unit waterlines.
- To assess the effectiveness of all the three disinfectants used at different concentrations in reducing the bacterial contamination of dental unit water.

## 4. Materials and Methods

Thirty dental units were selected for the study that was used most often for the dental treatments in Jaipur Dental College, Jaipur. Water samples were collected from 15 dental units each from the department of conservative and endodontics and the department of pediatric dentistry. None of the selected units had ever been treated for removal of biofilm or reduction of planktonic bacteria. Water samples between 10 and 50 mL were collected from the outlet of air/water syringe and high speed hand piece. Baseline samples were obtained at the start of the study. Samples were collected on Monday morning before the beginning of the working day.

Before sample collection, the end of each hand piece and two-way syringe was disinfected with 70% alcohol to avoid other sources of contamination. Water splashing was minimized when filling the sample container and any contact between the hand piece and the container was avoided. A volume of 10–50 mL of water was collected in sterile containers containing 0.1 gm 100 mL^−1^of sodium thiosulphate (Na_2_S_2_O_3_·5H_2_O) to remove residual chlorine. Samples were stored in a refrigerator and processed at the laboratory within two hours. The total viable count was estimated to assess the microbial contamination in the dental unit water line. The total viable count was then done to assess the microbial contamination in the dental unit water line.

The study was designed to determine the efficacy of different disinfectants at various concentrations on DUWLs. Thus each group of 10 dental chairs was treated with a particular disinfectant for a period of one week. Concentration was gradually increased to prevent carry-over effect of disinfectants. Cross disinfection was also avoided by treating a single dental chair with a similar disinfectant each time. Disinfection is a process that eliminates many or all pathogenic microorganisms on inanimate objects with the exception of bacterial endospores. 

Three disinfectants were diluted with distilled water to achieve 1 : 100, 1 : 10, 1 : 1 dilution and their effect in reducing the microbial contamination in the dental unit waterlines was studied.

1. Aloe vera (Sample A): commercially available aloe vera juice was used in the present study. The aloe vera juice was subjected to microbiological analysis before the start of the study to rule out the chances of contamination and to ensure the efficacy of the disinfectant. The aloe vera juice was found to be sterile and was therefore included. Sample A was diluted with distilled water to achieve concentrations of 1 : 100, 1 : 10 and 1 : 1.
2. 10% hydrogen peroxide (Sample B) was also diluted at 1 : 100, 1 : 10 and 1 : 1.
3. 5% sodium hypochlorite (Sample C) was similarly prepared to achieve 1 : 100, 1 : 10 and 1 : 1 dilution.

The three disinfectant Samples A, B, and C were added to the reservoir bottle of the 30 dental chairs from which baseline samples were obtained. Weekly disinfecting regimen was followed by adding 200 to 250 mL of disinfectant in the reservoir bottle that supplied the dental unit and the solution was run through the system for two minutes. The disinfectants were added on the weekend just before the commencement of that day's work and the unit was then turned off and the disinfectant was left in situ. Water samples of 10–50 mL from each treated unit's air/water syringe were collected in separate sterile containers under aseptic conditions and labelled before treating the first patient of the day and quantified for total viable counts.

It is a quantitative bacteriological analysis which enumerates total viable population capable of growing under a given set of conditions. Plate count is useful in determining the efficiency of water treatment. The plate count expresses the number of all colony forming bacteria in 1 mL of water. It provides information about the amount and type of organic matter in the water which may be useful indicating the efficiency of the processes used for water treatment or the suitability of water [6].

## 5. Results

The study included collection of water samples from each unit beginning with baseline collection and after DUWL exposure to disinfectant. Results obtained were the mean TVC in the treated water samples. When the different concentrations are compared, disinfectant property showed a direct relationship with the strength of the solution. The data was entered into a database using Microsoft Excel 2007 spreadsheet software, and the data was analyzed using common database and statistical software. Medians and frequencies of bacterial counts were calculated. The baseline total viable count for the thirty dental units of water line were ranging from 44 to 201 CFU/mL. The mean of all DUWL at baseline was 55.7 ± 33.6. The mean TVC of the three disinfectants irrespective of the dilutions of disinfectants were obtained (Table 1).

**Figure 1:**
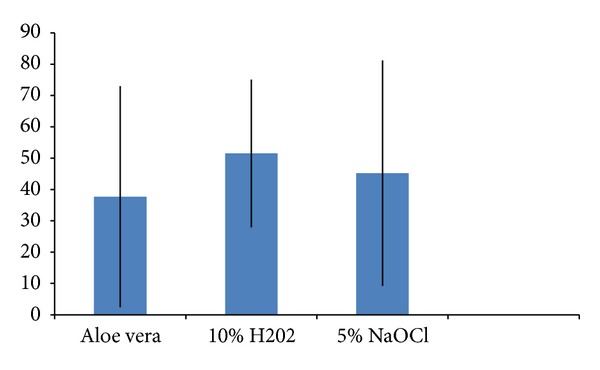
Reduction in mean TVC.

**Figure 2:**
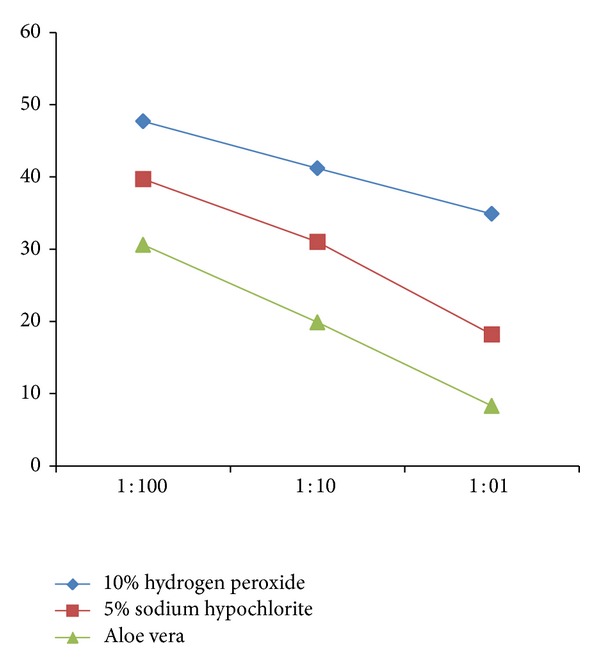
Comparison of three disinfectants at various concentrations (CFU/mL).

*Disinfection of Dental Unit Water Line (DUWL)*. Immediately after disinfection with sodium hypochlorite, hydrogen peroxide, and aloe vera, the output water from the disinfected dental chair units showed a high reduction in bacterial density (Figure 1). When relevance of change in dilution was analyzed, it was found that increase in strength reduces the total viable count (Table 3 and Figure 2).

**Figure 3:**
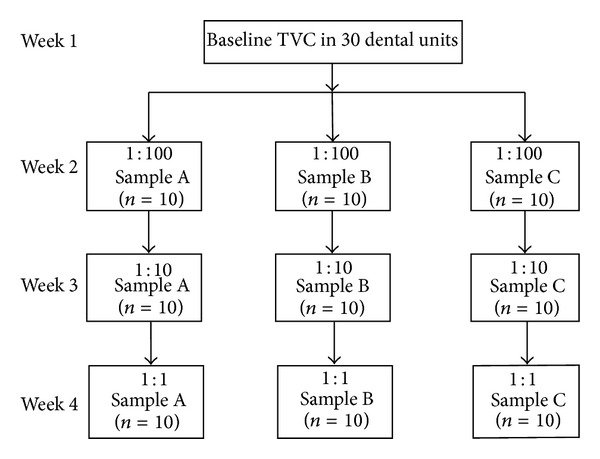
Standard plate count or total viable count (TVC).

No significant difference was observed when the three disinfectants were compared (*F* = 5.96, *P* > 0.01) (Figure 3). 

The difference was confirmed through one-way ANOVA where by increasing the concentration of aloe vera its disinfectant properties also increased (*F* = 2.9, *P* < 0.05) (Table 2).

## 6. Discussion

The biofilm on the inner surface of the tubing of dental units provides a continuous reservoir of microorganisms [7]. With the increasing number of immunocompromised and medically compromised individuals receiving regular dental treatment, contaminated dental unit output water poses a serious risk of infection. Not only patients but also dentists and dental professionals are at the risk of being infected with opportunistic pathogens such as *Pseudomonas* or *Legionella* species by means of cross infection or following aerosol formation from water emanating from DUWLs [8]. In this regard, effective practical methods for controlling microbial contamination of DUWLs need to be developed. 

High concentration of water borne organisms causes multiple public health problems. Contamination of water lines could be inhibited by using some disinfectants. Removal of these substances from water delivered into patient's mouth may reduce the potential for posttreatment inflammatory episodes [9]. The purpose of this study was to determine the extent of bacterial contamination of DUWLs in Jaipur Dental College and to compare the effect of aloe-vera-based disinfectant, 10% hydrogen peroxide, and 5% Sodium hypochlorite in reducing the bacterial density.

The tubing containing water is an ideal habitat for a host of microorganisms, leading to biofilm formation. The water travels through the tubing only for a few hours every working day and is enriched with organics and gets contaminated during operation. This ensures the growth of bacteria in the system, especially at the surfaces leading to the establishment of a stable source of inoculum for sterile water [10].

The ideal properties for an agent required to treat DUWL include low toxicity, low cost, ease of treatment, compatibility with a wide range of materials, and broad spectrum antimicrobial efficacy, especially against biofilms [11]. A disinfecting process should (i) kill bacteria in the water phase, (ii) kill biofilm-embedded cells, (iii) remove biofilm from the surface (as a “killed” biofilm can be a source of endotoxin and also allows rapid recolonization of a new and viable biofilm, which may occlude the tubing), (iv) be easily performed and offer continuous protection, thereby eliminating the root cause of poor dental unit water quality, and (v) provide continued efficacy during periods of nonuse, such as overnight and weekends. The disinfectant exhibiting these attributes would be easier to explain to patients and easier for practitioners to manage than the remedial treatment processes [7]. Considering the ideal properties of the disinfectant, herbal-based disinfectant like aloe vera was introduced which conforms with the antimicrobial properties. Aloe vera being a natural ingredient is said to be nontoxic and biodegradable. 

In the present study, the three disinfectants selected were found to be compatible with the DUWLs of the dental chair units used in the study. There was significant reduction in the mean CFU count when DUWLs were treated with disinfectants each for a period of one week. The results were in compliance with McEntegart and Clark [12] who proved the benefit of disinfectants for eliminating the CFU in routine use. However, the disinfectants should be evaluated for their effect on different pathogens and the duration of their effectiveness.

Aloe vera is found to have antimicrobial and antifungal properties. It consists of essential oil of *E. camaldulensis*, characterized by the presence of high concentrations of 1,8-cineole with well-documented antimicrobial activity [13]. Essential oils are capable of affecting biofilm formation. They significantly decrease bacterial adhesion and affect bacterial viability in biofilms [14]. The efficacy of aloe vera liquid as an antibacterial agent is shown to have a wide range of effectiveness against Gram-positive (Gram +ve) and Gram-negative (Gram −ve) bacteria due to an extract of the inner gel of the plant *Aloe barbadensis* Miller or *Aloe vera* (L.) [15]. The antimicrobial agents of aloe vera gel were reported to effectively kill or greatly reduce or eliminate the growth of *Streptococcus Aureus, Klebsiella pneumonia, Streptococcus pyogenes, Pseudomonas aeruginosa, E. coli, and Helicobacter pyroli* [16]. 

Whole leaf components are proposed to have direct antibacterial properties including anthraquinones and saponins, while polysaccharides have been attributed within direct bacterial activity through the stimulation of phagocytic leucocytes to destroy bacteria. Pyrocatechol is a hydroxylated phenol, known to be toxic to micro-organisms. The site and number of hydroxyl groups on the phenol group are thought to have relation with their toxicity to microorganisms and the increase in hydroxylation. The phenolic group present in aloe vera extracts acts by denaturing the proteins and cell membranes. They act as disinfectants and are effective in the presence of organic matter and remain active even long after application [16].

*Streptococcus pyogenes *and *Streptococcus faecalis *are two microorganisms that have been inhibited by aloe vera gel. Aloe gel is bacteriostatic or bactericidal against a variety of common wound-infecting bacteria in vitro: *Staphylococcus aureus, Streptococcus pyogenes, Serratia marcescens, Klebsiella pneumoniae, Pseudomonas aeruginosa, E. coli, Salmonella typhosa, *and *Mycobacterium tuberculosis* [17].

According to the study by Agarry et al. [18], *Aloe vera* gel and the leaf have inhibitory effect on *S. aureus *with zone of inhibition 18.0 and 4.0 mm, respectively. Among the bacteria and fungi tested, *A. vera *gel possesses greatest inhibitory effect on the *S. aureus. *Aloe gel is also used to promote wound healing due to the presence of some components like anthraquinones and hormones, which possess antibacterial, antifungal, and antiviral activities.

*Aloe vera* gel reportedly was bactericidal against *Pseudomonas aeruginosa *which is also among one of the microorganisms isolated from the DUWL. All the reported findings about the antimicrobial properties of aloe vera support the evidence of using aloe vera as a disinfectant in the present study. A processed *aloe vera* gel preparation reportedly inhibited the growth of *Candida albicans* [19].

The study was designed in such a way that the strength of disinfectant was subsequently increased after every week in the same dental chair. Treating a dental chair with single disinfectant would prevent error due to carry-over effect. Aloe vera was proved to have superior disinfecting properties as compared to 5% NaOCl and 10% H_2_O_2_ when used at various dilutions. The strength of each disinfectant was increased every week, starting with the lowest (1 : 00) and achieving the highest dilution (1 : 1). All the three disinfectants showed significant reduction in mean CFU/mL at various dilutions. Out of all, aloe vera was most effective at higher concentration (1 : 1).

Chemical compounds such as chlorhexidine gluconate, chlorhexidine acetate, sodium hypochlorite, povidone iodine, and Listerine have been advocated for use in DUWL. These are all complex molecules with varying degree of toxicity. The conventional disinfectants like sodium hypochlorite proves to be a problem as they are highly corrosive and the taste and smell are unacceptable. Aloe vera is suggested to be a potent antimicrobial agent as it was helpful in reducing the microbial contamination of dental unit water lines. Aloe vera was having superior properties when compared with hydrogen peroxide and sodium hypochlorite. It was also observed in the present research that with increasing the concentration the effect of disinfectant also increased.

### 6.1. General Recommendations

1. Chemical disinfectants can be replaced by aloe-vera-based disinfectants.
2. Aloe vera is proved to be a cost-effective disinfecting agent.
3. Use of chemicals will cause clogging and corrosion of dental tubings and airotors which can be prevented by using aloe-vera-based disinfecting agent.

### 6.2. Use and Care of Hand Pieces, Antiretraction Valves, and Other Intraoral Devices Attached to Dental Unit Water Lines

1. Routine sterilization procedures, that is, steam under pressure (autoclaving), dry heat, or heat/chemical vapor between-patient use is recommended for all high-speed dental hand pieces, low-speed hand piece components used intraorally, and reusable prophylaxis angles.
2. Internal surfaces of high-speed hand pieces, low-speed hand piece components, and prophylaxis angles often get contaminated during various dental procedures. This may result in cross contamination between patients.
3. Retraction valves in dental unit water lines may cause aspiration of patient material back into the hand piece and water lines; anti retraction valves should be installed to prevent fluid aspiration and to reduce the risk of transferring potentially infective material.
4. Routine maintenance of antiretraction valves is necessary to ensure effectiveness; the dental unit manufacturer should be consulted to establish an appropriate maintenance routine.
5. High-speed hand pieces should be run to discharge water and air for a minimum of 20–30 seconds after use on each patient. This procedure is intended to aid in physically flushing out patient material that may have entered the turbine and air or water lines.

## 7. Conclusion 

Improving the water quality from dental unit water lines is of considerable importance. Every effort should be made to eliminate not only planktonic bacteria but also the biofilm within the water lines. This prevents the risk of cross-infection amongst treated patients and the dental staff who are regularly exposed to contaminated water and aerosols generated from using dental hand pieces. The development of herbal biocide in the form of aloe vera will prove to be a revolution in disinfecting dental unit water lines. Further research is advocated to test the efficacy and shelf life of aloe vera not only in disinfecting the dental unit water but also to be applied in other areas for use as hospital disinfecting solution.

## 8. Key Messages

The quality of water in a dental unit is of considerable importance because patients and dental staff are regularly exposed to water and aerosol generated from the dental unit. The aim of this study was to evaluate the use of herbal-based disinfectant in reducing contamination of dental unit water line.

## Figures and Tables

**Table 1:** Mean TVC of the three disinfectants.

Disinfectant	Mean	SD
Aloe Vera (Sample A)	37.7	35.3
10% H_2_O_2_ (Sample B)	51.5	23.6
5% NaOCl (Sample C)	45.2	36.0

**Table:** (a)

	df	ss	mss	*F* value	*P* value	Significance
Treatment	2	3805.016667	1902.508333	5.96	0.00350	∗∗
Concentration	3	82616.166667	27538.722222	86.30	86.30	∗∗
Treatment* Concentration	6	4190.583333	698.430556	2.19	0.04950	∗
Error	108	34464.200000	319.112963			

**Table:** (b)

Comparison	Std. error	S.E. difference	*t* value 5%	C difference
Treatment	2.824504	3.994452	1.982127	7.917512
Concentration	3.261457	4.612396	1.982127	9.142356

**Table 3:** Comparison of three disinfectants at various concentrations (CFU/mL).

	10% hydrogen peroxide	5% sodium hypochlorite	Aloe vera
1 : 100	47.7	39.7	30.6
1 : 10	41.2	31	19.9
1 : 1	34.9	18.2	8.3
